# Low-Grade Glioma Clinical Trials in the United States: A Systematic Review

**DOI:** 10.3390/life14091133

**Published:** 2024-09-09

**Authors:** Emily Xu, Jonathan Patterson, Angelo Angione, Alexander Li, David W. Wu, Ebrar Akca, Omar Elghawy, Alexander Barsouk, Jonathan H. Sussman

**Affiliations:** 1 Department of Neurosurgery, Perelman School of Medicine, University of Pennsylvania, 3401 Civic Center Blvd., Philadelphia, PA 19104, USA; 2 Graduate Group in Genomics and Computational Biology, Perelman School of Medicine, University of Pennsylvania, Philadelphia, PA 19104, USA; 3 Director of Medical Oncology, Allegheny Valley Hospital, 1301 Carlisle St., Natrona Heights, PA 15065, USA

**Keywords:** low-grade glioma, clinical trial, healthcare disparities, race/ethnicity

## Abstract

Low-grade glioma (LGG) is a malignancy of the central nervous system that is often treatable with surgical resection and chemoradiation. However, despite an initial positive response to standard therapy, most LGG eventually progress to high-grade gliomas which are nearly uniformly fatal. There is a pressing need for more clinical trials and greater clinical trial accessibility to improve the standard of care of LGG to delay or prevent its progression. In this study, we systematically examined the scope and inclusion of clinical trials for LGG based in the United States. This cross-sectional study analyzes trends in trial design and reported demographic data from completed LGG trials registered on ClinicalTrials.gov between 2010 to 2023. Inclusion criteria, investigational therapies, primary outcomes, and preliminary results were compared and summarized. A total of 14 trials with 1067 participants were included in the study. Most of the trials were not exclusive to LGGs and 14% had mutation-specific inclusion criteria. To date, two of the trials have led to new FDA-approved treatments. All trials reported age and sex, while only 57% reported race and 43% reported ethnicity. Individuals identifying as Black or African American and Asian or Pacific Islander were statistically underrepresented. Lastly, we investigated the geographic distributions of trial sites across the United States, which demonstrated several coverage gaps in the Rocky Mountain and Southeast regions. These findings suggest specific areas for improvement in LGG clinical trial reporting and accessibility.

## 1. Introduction

Low-grade gliomas (LGGs) comprise a group of slow-growing tumors that arise from glial cells, including astrocytomas, oligodendrogliomas, and mixed gliomas. They comprise around 6% of all adult primary central nervous system (CNS) tumors [1,2] and predominantly affect young adults in the fourth decade [3]. They are defined by the World Health Organization (WHO) classification as Grades 1 and 2, but most eventually progress to high-grade glioma over time, which is nearly uniformly fatal [4]. The standard of care remains a combination of surgery, chemotherapy, and radiation [5]. Although many new therapeutics have been investigated in the past decades, LGG remains an incurable disease. For many patients, participation in a clinical trial can be the only remaining treatment option. Therefore, unequal access to trials may yield healthcare disparities. Diversity in clinical trials for LGG is also particularly important given that incidence rates and survival vary significantly with age, sex, race, and ethnicity [2]. These demographic differences in LGG epidemiology necessitate thoughtful study planning and results reporting in clinical trials. 

Increasing attention has turned to the need for clinical trial diversity in oncology [6,7]. Several studies have demonstrated gaps in inclusion in clinical trial enrollment of women, older adults, and minority racial and ethnic groups [8,9,10]. Hispanic patients made up 3% of oncology trials from 1996–2002 while representing 7% of cancer prevalence [11]. Similar disparities were also seen in Asian patients. In 2015, only 2.7% of oncology clinical trial participants identified as Black or African American [12], despite making up 9% of total U.S. cancer prevalence [13]. In that same time frame, 24% of psychiatric disorder trial participants were Black and African American, which is notable given that they constitute 10% of the national population with mental illness [14]. A lack of diversity among trial patients has significant consequences for the generalizability of the clinical findings and contributes to further under enrollment among those groups.

The aim of this study is to examine current demographic, geographic, and therapeutic modality trends in interventional LGG clinical trials that are registered in ClinicalTrials.gov, the largest public database of clinical trials. We identify disparities in patient recruitment and gaps in trial reporting. 

## 2. Materials and Methods

### 2.1. Data Collection 

**Figure 1:**
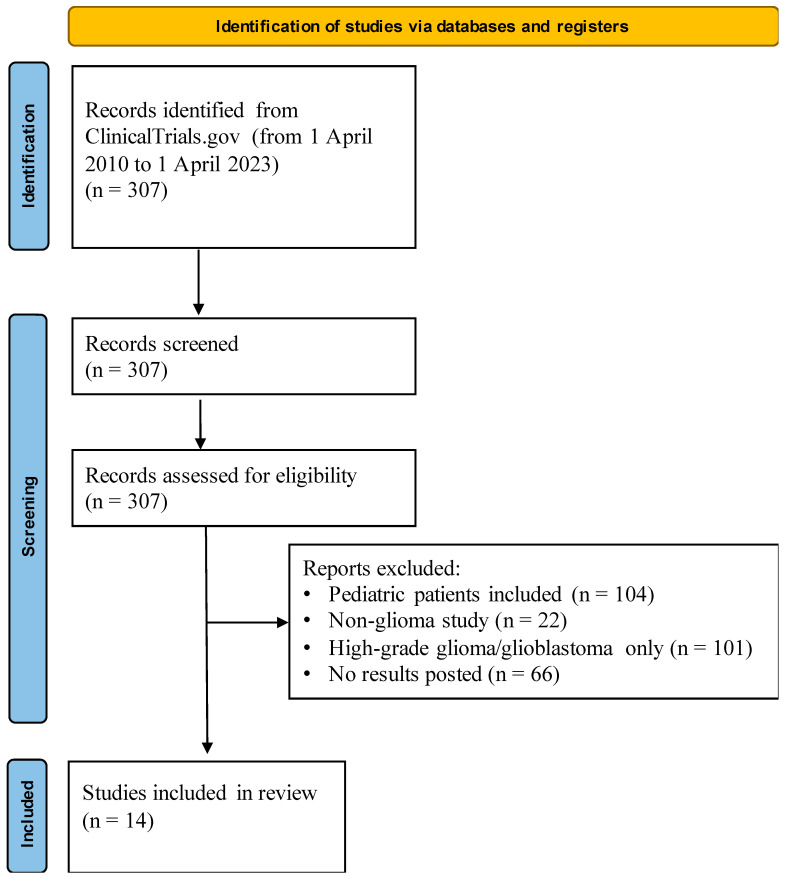
PRISMA flow diagram for identification of relevant low-grade glioma clinical trials.

Data were collected from all interventional United States-based adult low-grade glioma clinical trials registered on ClinicalTrials.gov (accessed on 1 June 2024) and completed between 1 April 2010, and 1 April 2023. The terms “Low-grade glioma”, “LGG”, “Grade I Glioma”, and “Grade II Glioma” were searched as “Condition/Disease” query items in the ClinicalTrials.gov database. The results were further refined to studies with only adults (18 years of age or older) and completed trials with reported results (Figure 1). Two independent reviewers (EX, JP) screened the studies for inclusion. Disagreement was resolved by a third reviewer (JHS). The final cohort for analysis yielded 14 studies with 1067 participants. Descriptive data from the trials, including funding sources, costs, partner institutions, and trial site information, as well as participant demographic data, such as race/ethnicity, sex, and age, were extracted. 

Total LGG incidence and mortality data were collected from the National Cancer Institute’s Surveillance, Epidemiology, and End Results (SEER) program [13]. The SEER database “SEER 17 Registries, Nov 2023 Sub (2000–2021)” was used to collect LGG cases from 2010 to 2021. The following ICD-O-3 codes allowed for the identification of LGGs: 9382/3, 9384/1, 9400/3, 9410/3, 9411/3, 9412/1, 9413/0, 9420/3, 9421/1, 9424/3, 9425/3, 9431/1, and 9450/3 [2]. Subjects < 20 years were excluded. A cohort of 7306 patients was obtained using all screening criteria.

This study followed the Preferred Reporting Items for Systematic Reviews and Meta-Analyses (PRISMA) reporting guidelines [15] and has not been registered in any database.

### 2.2. Trial Stratification and Analysis

Trials were stratified into “public”, “private”, or “mixed” institution trials. “Public institutions” were defined as hospitals associated with the Department of Veteran’s Affairs, a public university system (e.g., University of Pittsburg Medical Center), or the National Institutes of Health (NIH) Clinical Center. All other institutions were defined as “private institutions”. Trials with both a “public” and “private” institution were classified as “mixed”. Trials were stratified by investigational modality (i.e., drug, radiation, surgical procedure, etc.), year, and trial phase. Race and ethnicity were categorized according to the categories established by the Office of Management and Budget (OMB) Standards for the Classification of Federal Data on Race and Ethnicity [16]. 

### 2.3. Statistical Analysis

All statistical analyses and graphics were generated in R (v4.3.1, R Foundation for Statistical Computing, Vienna, Austria). Average values were reported with standard deviation. Two-proportion z-tests were used to compare percentage data. Statistical significance was defined as *p*-values < 0.05. 

## 3. Results

### 3.1. Overview of Low-Grade Glioma Trials 

#### Inclusion Criteria

Only 4 out of the 14 trials were exclusive to LGGs, while the other trials also included HGGs (Table 1). Two trials were mutation-specific; one focused on IDH1-R132H Grade 2 glioma, while the other mutation-specific trial included tumors that had a BRAF-V600E mutation (NCT02034110). The BRAF trial also included non-CNS tumors, such as thyroid cancer, biliary tract cancer, gastrointestinal cancer, and hematologic cancers. The other trial to include non-CNS tumors was NCT00492089, which also included head and neck cancer. The only diagnosis-specific trials were two studies focused on recurrent ependymomas. These were among the four total trials that only included recurrent disease. 

### 3.2. Interventions and Primary Outcomes

The most common therapeutic type investigated in the trials was a drug (78.6%), followed by a biologic (21.4%) and radiation (7.1%) (Table 2). Two out of the 14 trials investigated a novel agent. Four trials studied multiple investigational agents, while two trials examined multimodal interventional strategies. The interventions in the 14 LGG trials either were targeted to treating the brain tumor itself or treating the side effects of treatment (Table 1). Though no trials had surgical intervention, one did investigate a drug, 5-aminolevulinic acid (5-ALA), to improve surgical resection. 

The trials focused on glioma therapy covered all three tenets of the current treatment paradigm—surgery, radiation, and systemic therapy. Three of the trials included temozolomide, an alkylating agent with FDA approval for the treatment of anaplastic astrocytoma and glioblastoma [17]. It was studied as a stand-alone drug and in combination with either radiotherapy or lapatinib, which is a tyrosine kinase inhibitor that targets epidermal growth fractor receptor (EGFR) and Herceptin-2 (HER2). Lapatinib is currently approved for treating advanced or metastatic breast cancer [18]. Three other trials investigated kinase inhibitors including everolimus (mTOR inhibitor), a combination of dabrafenib (BRAF inhibitor) and trametinib (MEK inhibitor), and erlotinib (EGFR inhibitor). Three trials included a biologic as a therapeutic strategy for tumor treatment. One studied the combination of carboplatin with bevacizumab, a monoclonal antibody blocking vascular endothelial growth factor (VEGF), for recurrent ependymoma. The other two trials were vaccines. One featured autologous dendritic cells pulsed with tumor lysate and the other investigated an IDH1 peptide vaccine. Other than systemic treatment, one trial focused on studying proton radiation and whether its side effect profile was improved compared to standard radiation therapy. Finally, one trial aimed at improving the surgical aspect of glioma treatment investigated 5-ALA, a fluorescent agent that can highlight tumor tissue during surgery. The most common primary outcome measured for trials in this category was progression-free survival (*n* = 6, 54.5%). 

The remaining three trials were focused on treating the side effects of tumor treatment, namely radiation-related CNS effects. They investigated bevacizumab, donepezil, and armodafinil, respectively. Donepezil is an acetylcholinesterase inhibitor approved for dementia treatment while armodafinil is a dopamine reuptake inhibitor used for narcolepsy and obstructive sleep apnea. The primary outcomes for trials in this group were varied including radiographic response, cognitive function, and participant retention. 

#### Preliminary Results

To date, nine trials have reported meeting the primary clinical endpoint and published a full analysis of the trial cohort [19,20,21,22,23,24,25,26,27]. Furthermore, two trials have resulted in FDA approval. Based on the results of NCT02034110 (ROAR) and NCI-MATCH trials, the combination of dabrafenib and trametinib received FDA approval in June 2022 for any BRAF-mutated solid tumors [19]. Additionally, NCT01116661 was terminated early due to efficacy and ALA received FDA approval for intraoperative tissue imaging [20]. 

### 3.3. Demographic Recruitment and Reporting

#### 3.3.1. Trial Characteristics

In the final cohort of 14 studies, only 3 (21.4%) were reported as randomized (Table 2). Most (71.4%) were Phase 2 trials and the rest of the trials were evenly split between Phase 1 (7.1%), Phase 1/2 (7.1%) and Phase 3 (7.1%). Three trials had a two-arm design, while 11 had one arm. The average enrollment was 76.2 ± 68.5 participants. Trials were conducted at single (57.1%) and multiple (42.9%) testing sites. All the trials that had multiple testing sites were across multiple states. Two trials included international testing sites as well. Regarding investigational institution type, an equal proportion of the trials were located exclusively at private institutions as public institutions (Table 2). 

#### 3.3.2. Race and Ethnicity 

Over half (57.1%) of the trials reported the race of participants and 42.9% reported race consistent with Office of Management and Budget (OMB) standards (Table 2). When compared to national incidence rates of LGG by race, LGG trials underrepresented Black or African American (3.7% versus 5.8%, *p* = 0.03) and Asian or Pacific Islander (3.7% versus 7.4%, *p* = 0.0002) patients. American Indian/Alaska Native patients were also underrepresented by a factor of 5 (0.2% versus 1.0%), although was not large enough to reach statistical significance (*p* = 0.051). There was also a relative lack of Hispanic or Latino patients (2.6% versus 16.8%, *p* < 0.0001). Trials held at private institutions reported race 80% of the time, while trials held at public institutions reported race 40% of the time. There were no significant differences in the percentage of non-White participants based on whether the trials were conducted at a private or public institution (*p* = 0.69). After comparing LGG trials in 2012–2017 to those in 2018–2023, there were no differences in the percentage of non-White (7.54% vs. 6.67%, *p* = 0.71) or Hispanic participants (2.31% vs. 3.37%, *p* = 0.58). 

#### 3.3.3. Sex and Age

All trials reported the sex and age breakdown of their participants (Table 2). There were no significant differences in sex between the trial participants and the broader LGG population in the U.S. (*p* = 0.11, Table 3). 

#### 3.3.4. Geographic Diversity

**Figure 2:**
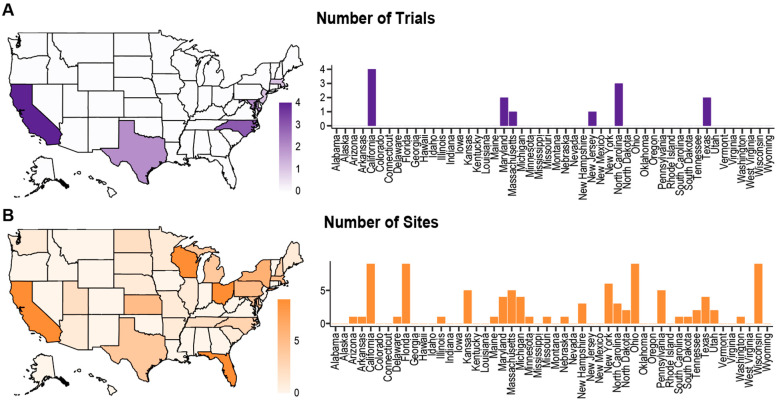
**Geographic distribution of low-grade glioma clinical trials across the United States.** Map and bar graph of (**A**) independent clinical trials and (**B**) trial sites conducted in each state. The shading of each state represents the number of clinical trial sites hosted within its borders.

The geographic distribution of trial sites was analyzed by the U.S. state. There were 27 different states that had at least one LGG trial site (Figure 2). The states with the greatest number of trials were California, Florida, Ohio, and Wisconsin, which each had nine sites. There was a dearth of sites in the Rocky Mountain region and in most states in the Southern U.S. There also were no sites in Hawaii or Alaska. 

## 4. Discussion 

In this cross-sectional study, we examined the state of LGG clinical trials in the United States. Despite the low number of completed studies, we found a broad range of therapeutic strategies and highlighted a significant portion of studies that focus on quality of life for treated patients. We also examined participant demographics and compliance with reporting guidelines. We identified an underrepresentation of several minority groups, including those identifying as Black or African American, Asian or Pacific Islander, and Hispanic and Latino. Furthermore, we identified gaps in reporting and reporting consistency. 

The first striking observation was the paucity of trials investigating LGG, especially when compared to HGG trials. We were only able to include 14 trials from the previous decade that explicitly included low-grade gliomas in their inclusion criteria. In contrast, our previous cross-sectional survey of HGG trials during the same period identified 201 studies [28]. There are many unique challenges facing LGG clinical trials when compared to HGG trials. LGGs are relatively infrequent, which can limit participant recruitment and necessitate multi-center studies for larger phase 2 and 3 trials. Additionally, because of the slow-growing nature of the disease and longer-term recurrence, follow-up time needs to be longer than 5 years to detect treatment effects, which presents difficulties with extended funding [29]. Furthermore, because of the relatively better overall survival and positive initial response to treatment, federal grant applications for therapeutic LGG trials may be less competitive than trials for HGG therapeutics. Therefore, many trials, such as those for IDH immunotherapies, have only been studied in low-grade tumors that have transformed into higher-grade tumors (NCT02454634, NCT02968940). 

Given that there are so few completed trials focused on low-grade gliomas, representation becomes even more crucial. Women and minorities are required to be included in clinical trials by the NIH Revitalization Act [30]. Reporting guidelines have since been further updated so that there are minimum reporting standards for race/ethnicity [16] and that sub-group analyses must be completed based on race/ethnicity [31]. Despite widespread efforts, the representation of some minority groups in clinical trials has yet to significantly improve [32]. Our study finds that groups identifying as Black or African American and Asian or Pacific Islander are underrepresented in trials when compared to the national incidence of LGG within those populations. The number of Hispanic or Latino patients was also disproportionately low among the patient participants. We also did not observe a trend of increasing inclusion in later trials, which is largely consistent with the findings of other analyses of cancer clinical trials [8]. The lack of diversity may be due to the predominance of early-phase trials in our cohort. Earlier phase trials are subject to lower enrollment, stricter resource constraints, and high failure rates, which may lead researchers to underprioritize diverse recruitment [33]. However, these gaps must also be contextualized by the lack of reporting of race and ethnicity characteristics in many of the trials. Having complete and standardized data is crucial to identifying and understanding the drivers of health disparities so that interventions can be tailored to specific problems [9,34,35]. 

We also analyzed the geographic diversity in clinical trial sites and found several regions that had no sites and therefore no access to participation. The concentration of sites in particular regions of the country may contribute to the racial and socioeconomic disparities. One institutional study that evaluated various factors that impacted clinical trial screening and enrollment found that minorities enrolled in clinical trials lived on average 30 miles closer to the cancer center when compared to those who did not enroll [36]. Another research study found that the most common reason patients chose not to participate in a trial was travel distance [37]. There have been several efforts by the National Cancer Institute to expand access to trials to underserved communities, including the development of the National Cancer Institute Community Oncology Research Program (NCORP) [38]. 

Given these findings, this study has several limitations. We only analyzed U.S.-based trials that were registered in ClinicalTrials.gov. Consequently, we were limited to the data that was reported on this website, which had inconsistent data reporting formats. For example, we could not analyze the age of participants, given the heterogenous statistics that were reported (mean, median, under/over age 65, etc.). Additionally, we did not include any trials that were registered internationally. Further work analyzing trial registries abroad, such as the European Union Clinical Trials Registry and the Chinese Clinical Trial Registry, may yield a more complete picture of the current status of LGG research. It may also yield common patterns of research interest, such as in temozolomide (EORTC 22033-26033). We were also unable to analyze the relationship between socioeconomic status and enrollment as no data regarding median household income were reported in any of the trials. Given that race and socioeconomic status are correlated, we were unable to disentangle which factor was more salient in affecting trial enrollment, which would have relevance in tailoring specific solutions or guidelines to increasing representation. Finally, the limited number of studies that were included constrained the analysis over time. 

## Figures and Tables

**Table 1:** Complete overview of low-grade glioma clinical trials in ClinicalTrials.Gov.

Clinical Trial Number	Study Title	Interventional Agent Investigated	Primary Outcome	Sponsor	Number of Participants	Number of Recruitment Sites	Results Submitted Year	Phase	Funder Type
NCT00492089	Bevacizumab in Reducing CNS Side Effects in Patients Who Have Undergone Radiation Therapy to the Brain for Primary Brain Tumor, Meningioma, or Head and Neck Cancer	Bevacizumab	Radiographic response rate (>25% Reduction in T2 Flair) at 6 weeks post-treatment	National Cancer Institute (NCI)	11	1	2012	Phase 2	NIH
NCT00369785	Donepezil in Treating Patients Who Have Undergone Radiation Therapy for Brain Tumors	Donepezil hydrochloride	Memory as measured by the Hopkins Verbal Learning Test (HVLT)	Wake Forest University Health Sciences	198	16	2015	Phase 3	Other
NCT01032200	Armodafinil in Treating Fatigue Caused By Radiation Therapy in Patients With Primary Brain Tumors	Armodafinil	Retention of participants 4 weeks post-radiotherapy	Wake Forest University Health Sciences	54	1	2016	Phase 2	Other
NCT00114140	Temozolomide and Radiation Therapy in Treating Patients With Gliomas	Temozolomide and Radiation Therapy	Overall Survival Rate at 3 Years Progression-free survival time Quality of life as measured by the Functional Assessment of Cancer Therapy Scale With Brain Module (FACT-BR) Phonemic verbal fluency as measured by the Controlled Oral Word Association test	Radiation Therapy Oncology Group	136	47	2017	Phase 2	Network
NCT00823459	Everolimus in Treating Patients with Recurrent Low-Grade Glioma	Everolimus	Progression-free survival at 6 Months	Susan Chang	58	1	2017	Phase 2	Other
NCT00045110	Erlotinib in Treating Patients with Recurrent Malignant Glioma or Recurrent or Progressive Meningioma	Erlotinib hydrochloride	Dose Limiting Toxicity (DLT) Maximum Tolerated Dose (MTD) 6 Months Progression-free survival	National Cancer Institute (NCI)	136	7	2017	Phase 1/2	NIH
NCT00681473	Late Effects of Proton Radiation Therapy in Patients with Low-Grade Glioma	Proton Radiation Therapy	Number of participants with late effects >3 months post radiotherapy	Massachusetts General Hospital	20	1	2017	NA	Other
NCT00313729	Temozolomide in Treating Patients With Low-Grade Glioma	Temozolomide	Response rate at 1 year	University of California, San Francisco	120	1	2018	Phase 2	Other
NCT01116661	Safety Study of Aminolevulinic Acid (ALA) to Enhance Visualization and Resection of Tumors of the Brain	5-Aminolevuline Acid (ALA)	Percentage of biopsies with tumorous content	University of California, San Francisco	199	1	2018	Phase 2	Other
NCT00826241	Dose-Dense Temozolomide + Lapatinib for Recurrent Ependymoma	Temozolomide and Lapatinib	Time to Progression up to 4 years	National Institutes of Health Clinical Center (CC)	58	6	2019	Phase 2	NIH
NCT01635283	Vaccine for Patients With Newly Diagnosed or Recurrent Low-Grade Glioma	Tumor lysate-pulsed autologous dendritic cell vaccine	Progression-free Survival (PFS) up to 44 months	Jonsson Comprehensive Cancer Center	5	1	2019	Phase 2	Other
NCT01295944	Carboplatin and Bevacizumab for Recurrent Ependymoma	Carboplatin and Bevacizumab	Progression-free Survival (PFS) after 1 year	National Cancer Institute (NCI)	35	2	2021	Phase 2	NIH
NCT02193347	IDH1 Peptide Vaccine for Recurrent Grade II Glioma	PEPIDH1M vaccine	Toxicity rate	Katy Peters, MD, PhD	24	1	2021	Phase 1	Other
NCT02034110	Efficacy and Safety of the Combination Therapy of Dabrafenib and Trametinib in Subjects With BRAF V600E- Mutated Rare Cancers	Dabrafenib and Trametinib	Overall response rate up to 92 months post-treatment	Novartis Pharmaceuticals	13	41	2022	Phase 2	Industry

**Table 2:** Summary characteristics of low-grade glioma clinical trials in ClinicalTrials.gov.

Characteristic	Number of Trials (% of All Trials), *n* = 14
**Intervention Type**	
Drug	11 (78.6%)
Radiation	1 (7.1%)
Surgical	0 (0%)
Biologic	3 (21.4%)
Other	0 (0%)
Utilizes Multiple Investigational Agents	4 (28.6%)
Utilizes a Multimodal Interventional Strategy	2 (14.3%)
Randomized	3 (21.4%)
**Testing Site Number**	
Single Testing Site	8 (57.1%)
Multiple Testing Sites	6 (42.9%)
**Testing Site Location**	
Testing Sites in Multiple States	6 (42.9%)
Multiple Testing Sites Across Single State	4 (28.6%)
Includes International Testing Sites	2 (14.3%)
**Investigational Institution Type**	
Private Investigational Institution	5 (35.7%)
Public Investigational Institution	5 (35.7%)
Mixed Investigational Institution	4 (28.6%)
**Demographics Reported**	
Reports Race	8 (57.1%)
Reports Race Consistent with OMB Standards	6 (42.9%)
Reports Ethnicity	6 (42.9%)
Reports Sex	14 (100%)
Reports Age	14 (100%)
**Phase**	
Phase 1	1 (7.1%)
Phase 1/2	1 (7.1%)
Phase 2	10 (71.4%)
Phase 3	1 (7.1%)
**Funding Source**	
NIH Funded	4 (28.6%)
Industry Funded	1 (7.1%)
Network Funded	1 (7.1%)
Other Funder	8 (57.1%)

**Table 3:** Comparison of patient race/ethnicity and sex in registered LGG clinical trials with total U.S. LGG patients.

Characteristic	Number of Trial Participants (% of Reported) *n* = 1067	Number of LGG Patients in U.S. (%) *n* = 7306	*p*-Value
**Race**			
White	558 (92.7%)	6219 (85.9%)	<0.00001 *
Black or African American	22 (3.7%)	418 (5.8%)	0.032 *
Asian or Pacific Islander	20 (3.3%)	536 (7.4%)	0.00016 *
American Indian or Alaska Native	1 (0.2%)	69 (1.0%)	0.051
Other/Not Reported	466	64	
**Ethnicity**			
Hispanic or Latino	10 (2.6%)	1228 (16.8%)	<0.00001 *
Not Hispanic or Latino	382 (97.4%)	6078 (83.2%)	
Not Reported	685	-	
**Sex**			
Male	575 (54.3%)	4154 (56.9%)	0.11184
Female	483 (45.7%)	3152 (43.1%)	

Significance was assessed using a two-proportion z-test. * indicates significance with *p* < 0.05.

## Data Availability

Any additional data and code used to generate the analysis and figures in the current study are available from the corresponding author upon reasonable request.

## Abbreviations

CNS, central nervous system; HGG, high-grade glioma; LGG, low-grade glioma; OMB, Office of Management and Budget; WHO, World Health Organization.
